# Genetic Diversity, Admixture, and Selection Signatures in a Rarámuri Criollo Cattle Population Introduced to the Southwestern United States

**DOI:** 10.3390/ijms26104649

**Published:** 2025-05-13

**Authors:** Maximiliano J. Spetter, Santiago A. Utsumi, Eileen M. Armstrong, Felipe A. Rodríguez Almeida, Pablo J. Ross, Lara Macon, Eugenio Jara, Andrew Cox, Andrés R. Perea, Micah Funk, Matthew Redd, Andrés F. Cibils, Sheri A. Spiegal, Richard E. Estell

**Affiliations:** 1 Department of Animal and Range Sciences, New Mexico State University, Las Cruces, NM 88003, USA; sutsumi@nmsu.edu (S.A.U.); arcox@nmsu.edu (A.C.); arperea@nmsu.edu (A.R.P.); funkm@nmsu.edu (M.F.); 2 Unidad de Genética y Mejora Animal, Departamento de Producción Animal, Facultad de Veterinaria, Universidad de la República, Montevideo 10129, Uruguay; eileen.armstrong@gmail.com (E.M.A.); eugeniojara19@gmail.com (E.J.); 3 Facultad de Zootecnia y Ecología, Universidad Autónoma de Chihuahua, Chihuahua 31453, Mexico; frodrigu@uach.mx; 4 Inguran LLC Dba STgenetics, Navasota, TX 77868, USA; pablo.ross@stgen.com; 5 USDA Agricultural Research Service Jornada Experimental Range, Las Cruces, NM 88003, USA; lara.macon@usda.gov (L.M.); sheri.spiegal@usda.gov (S.A.S.); 6 Dugout Ranch/Canyonlands Research Center, The Nature Conservancy, Monticello, UT 84535, USA; matthew.redd@tnc.org; 7 USDA Southern Plains Climate Hub, Oklahoma and Central Plains Agricultural Research Center, El Reno, OK 73036, USA; andres.cibils@usda.gov

**Keywords:** Creole cattle, heritage genetics, single nucleotide polymorphisms, population structure, positive selection

## Abstract

Rarámuri Criollo (RC) cattle have been raised by the isolated Tarahumara communities of Chihuahua, Mexico, for nearly 500 years, mostly under natural selection and minimal management. RC cattle were introduced to the United States Department of Agriculture-Agricultural Research Service Jornada Experimental Range (RCJER) in 2005 to begin evaluations of beef production performance and their adaptation to the harsh ecological and climatic conditions of the Northern Chihuahuan Desert. While this research unveiled crucial information on their phenotypic plasticity and adaptation, the genetic diversity and structure of the RCJER population remains poorly understood. This study analyzed the genetic diversity, population structure, ancestral composition, and selection signatures of the RCJER herd using a ~64 K SNP array. The RCJER herd exhibits moderate genetic diversity and low population stratification with no evident clustering, suggesting a shared genetic background among different subfamilies. Admixture analysis revealed the RCJER herd represents a distinctive genetic pool within the Criollo cattle breeds, with significant Iberian ancestry. Selection signatures identified candidate genes and quantitative trait loci (QTL) for traits associated with milk composition, growth, meat and carcass, reproduction, metabolic homeostasis, health, and coat color. The RCJER population represents a distinctive genetic resource adapted to harsh environmental conditions while maintaining productive and reproductive attributes. These findings are crucial to ensuring the long-term genetic conservation of the RCJER and their strategic expansion into locally adapted beef production systems in the USA.

## 1. Introduction

The first Criollo cattle populations trace their origins to Iberian cattle brought by Spanish explorers in the late 15th century [1]. Subsequent introductions between the 16th and 18th century included Iberian and West African cattle that are believed to have been brought to the Caribbean and Brazil [2]. These early introductions contributed to the genetic background of the American Criollo cattle, influenced by a combination of Spanish, Portuguese, and African breeds. The importation of European breeds in the 19th century and Indicine breeds in the 20th century further shaped the mixed ancestry of Criollo cattle [3,4,5,6]. Criollo cattle rapidly spread across the American continent, from the Great Plains of North America to the mountain ranges and desert plains of Patagonia [1,6,7]. Since their introduction to the Americas, most Criollo cattle breeds have undergone five centuries of development with minimal or no human intervention. Natural selection has provided Criollo cattle with remarkable adaptations to harsh environmental conditions, making it a unique resource for understanding the genetic mechanisms underlying such physiological, nutritional and behavioral adaptations [8]. Efforts were also made to preserve and expand this valuable genetic resource [4,9,10].

At least 33 distinct Criollo breeds have been recognized across the Americas [11]. Among them, the Rarámuri Criollo (RC) stands out as a unique biotype preserved by the Tarahumara communities, also known as Rarámuri or ‘Barefoot’ long-distance travelers [8,11]. The RC developed for approximately 500 years with minimal human management, largely shaped by natural selection in the isolated areas of the Sierra Tarahumara (Copper Canyon) in southwestern Chihuahua, Mexico [12]. The topography and climate characteristics of the Sierra Tarahumara region are diverse and extremely variable [13]. Steep terrain along with low ambient temperatures are predominant in the high-elevation Sierra Tarahumara, while high temperature extremes are common in the lower canyons and valleys of the subtropical region of the Sierra Tarahumara [13]. The harsh environmental conditions of the Sierra Tarahumara are further compounded by woody plant encroachment and the presence of a sparse herbaceous understory [8,11,14].

To evaluate their adaptation to hot desert rangelands, three RC bulls and 30 heifers were introduced to the USDA-ARS Jornada Experimental Range (JER) in southwestern New Mexico, USA, in 2005 [8,15]. These cattle belonged to isolated local families inhabiting the canyons of the lower Sierra Tarahumara, located between 200 and 400 m above sea level, and characterized by high ambient temperatures during the summer [8]. The RC from JER (RCJER) are a medium-sized animal, with cows and bulls weighing approximately 390 kg and 620 kg, respectively, and a birth weight of calves of approximately 21 kg. Both mature females and males have lyre-shaped horns and a wide variety of coat colors, including solid, brindle, and spotted patterns [16].

Previous research has identified important phenotypic traits that can explain the plasticity and high adaptation of RCJER cattle to desert rangelands [15]. The RCJER cattle adjust their grazing behavior seasonally, traveling longer distances and covering wider areas compared to commercial breeds when forage availability is limited [17,18,19,20]. The RCJER cattle have lower body temperatures than commercial beef cows, spending more time grazing and traveling during the hottest hours of the day [21], suggesting foraging plasticity and heat tolerance during summer.

Anecdotal [8,15] and experimental [22] observations reported that, compared to Angus x Hereford crossbreds, RC cattle consume more shrubs and other non-grass plants, such as honey mesquite (*Prosopis glandulosa* Torrey) and soaptree yucca (*Yucca elata*), minimizing grazing pressure of black grama (*Bouteloua eriopoda*), a highly preferred perennial grass of high conservation value in southwestern New Mexico, USA [18,22]. Additionally, large home ranges, diet breadth and heat tolerance may provide the RCJER with beneficial environmental and production outcomes, supporting local beef supply chains [23,24,25].

Previous findings indicated that RC cattle may enable ranchers to balance livestock production with rangeland conservation in the southwest USA and similar regions. While a growing number of studies have evaluated the conformation characteristics and phenotypic traits of RC cattle, information on the genetic structure is limited [14]. Understanding the ancestry and genetic composition of the RC cattle population is essential to document their genetic diversity and structure [26]. This information is critical for guiding genetic conservation programs and developing strategies for expanding RC cattle genetics into commercial beef cattle herds. Therefore, the aims of this study were (i) to assess genetic diversity, population structure, and ancestry, and (ii) to identify signatures of selection in the RCJER cattle population. Overall, this study provides a comprehensive genetic characterization of the RCJER population, including comparative analyses with other Criollo cattle breeds.

## 2. Results

### 2.1. Genetic Diversity

After quality filtering, the dataset consisted of 53,750 SNPs and 151 individuals; one animal was removed due to missing genotype data. The mean observed heterozygosity (H_O_) was slightly higher (0.371 ± 0.143; range: 0.007–0.662) than the expected heterozygosity (H_E_; 0.362 ± 0.136; range: 0.02–0.5).

To optimize the runs of homozygosity (ROH) detection, minor frequency allele (MAF) and linkage disequilibrium (LD) pruning were not applied [27]. After quality filtering, 56,709 SNPs and 151 individuals were retained. A total of 12,047 ROH segments were identified, with an average of 190.5 ± 14.45 segments per animal. Segments ranging from >1 to 2 Mb were the most prevalent, accounting for 63.25% of all ROHs, while segments > 8 Mb represented 8.17% (Table 1).

The genomic inbreeding coefficient based on ROH (F_ROH_) per animal ranged from 0.033 to 0.409, with a mean of 0.115. This wide range reflects significant variability in F_ROH_ values within the herd. Most values were around 0.1, indicating a high proportion of individuals with moderate inbreeding. However, some values exceeding 0.3 suggest the presence of a few individuals with elevated inbreeding levels. Across different segment lengths, F_ROH_ values ranged from 0.002 to 0.402, with the highest value observed in the > 1–2 Mb category (Table 1).

The historical effective population size (Ne) was analyzed using two different programs, SNeP and GONE. The SNeP program revealed a smooth decline in Ne over the past 121 generations, with an estimated value of 97 in generation 13 (Figure S1a). In contrast, the GONE program indicated a non-linear pattern in Ne estimation (Figure S1b). A steady decline in Ne was observed from generation 121 to 88, followed by a linear increase until generation 20 and subsequent steady decline until generation 9. Thereafter, Ne declined markedly, reaching a value of 33 for the most recent generation. Although the Ne estimates from SNeP and GONE differed qualitatively and quantitatively, both detected a declining trend in Ne towards the most recent generations.

### 2.2. Population Structure and Genetic Relationships

**Figure 1:**
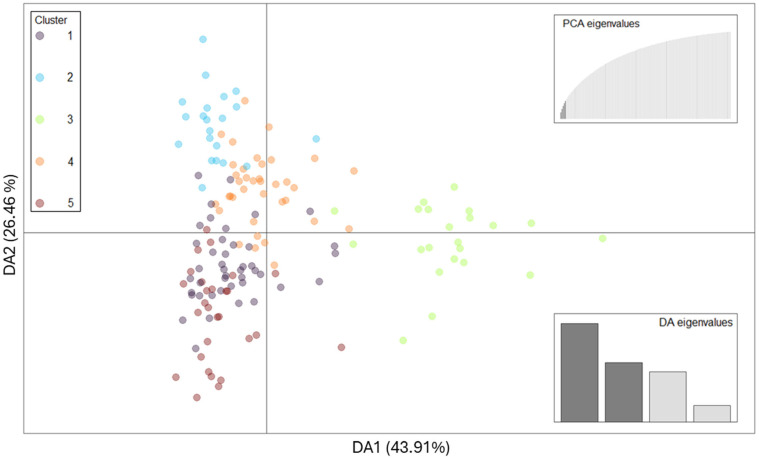
Discriminant analysis of principal components scatter plot of the first two discriminant axes of Rarámuri Criollo cattle from the Jornada Experimental Range. Five principal components (PCA; top right) and four discriminant axes (DA; bottom right) were retained. Dots represent individuals and the clusters are presented in different colors.

After data quality control, the dataset used to evaluate population structure consisted of 53,750 SNPs and 151 individuals. The principal component analysis (PCA) revealed that the RC individuals were scattered without a strong clustering or subgrouping (Figure S2). Therefore, a discriminant analysis of principal components (DAPC) was performed to further explore the genetic structure of the RCJER cattle population. The most likely number of clusters (K), determined using the K-means algorithm (K ranging from 1 to 10), was found to be five (Figure S3). Most of the clusters overlapped, indicating limited discrimination among them. However, some exceptions were observed in clusters 2 and 3, where distinct individuals were positioned at the extremes (Figure 1).

The kinship coefficient estimated by the KING-robust method [28] ranged from −0.291 to 0.439, with a mean of −0.002. Most values were close to or below zero, corresponding to unrelated animals. As expected, some pairwise comparisons showed values > 0.125, indicative of a second-degree relationship, and a few pairwise comparisons had a value > 0.25, indicative of a first-degree relationship.

### 2.3. Relationship with Other Criollo Cattle Populations

The final dataset used to evaluate the relationship between RC and other Criollo cattle populations consisted of 10,612 common SNPs and 105 individuals (Table S1).

**Figure 2:**
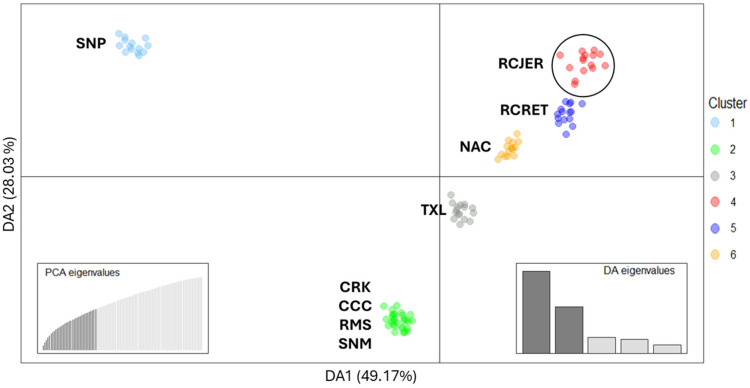
Discriminant analysis of principal components scatter plot of the first two discriminant Axes of nine Criollo cattle populations. Thirty-five principal components (PC; bottom left) and five discriminant axes (DA; bottom right) were retained. Dots represent individuals and the clusters are presented in different colors. Rarámuri Criollo from the Jornada Experimental Range are highlighted within a circle. RCJER: Rarámuri Criollo cattle from Jornada Experimental Range, RCRET: Rarámuri Criollo cattle from Rancho Experimental Teseachi, TXL: Texas Longhorn, NAC: North American Corriente, CRK: Florida Cracker, SNP: Senepol, CCC: Costeño con Cuernos, RMS: Romosinuano, SNM: San Martinero.

The PCA revealed that the Criollo populations formed distinct clusters, suggesting genetic differentiation among them (Figure S4). The relationship among Criollo populations was further explored using DAPC analysis. The most likely number of K was 6 (K ranging from 1 to 15; Figure S5). All six clusters were clearly distinguishable, with each corresponding to a single Criollo breed, except for cluster 2, which included four Criollo breeds adapted to humid tropical conditions: Florida Cracker (CRK), Costeño con Cuernos (CCC), Romosinuano (RMS), and San Martinero (SNM) (Figure 2).

Both principal component 1 (PC1) and discriminant axis 1 (DA1) clearly differentiated the three North American populations, RC, North American Corriente (NAC) and Texas Longhorn (TXL), which are adapted to the dry and hot conditions of the region. These cattle populations diverged from the remaining Criollo populations, which are better adapted to the humid tropical conditions of Central America, the Caribbean, and Colombia in South America. The PC2 and DA2 further separated the two RC populations from TXL and NAC. As expected, the RCJER was positioned relatively close to the RC cattle belonging to the Rancho Experimental Teseachi (RCRET) in Chihuahua, Mexico.

The fixation index (F_ST_) pairwise distances (Figure S6) supported the PCA and DAPC results. The F_ST_ distances between RCJER and most populations were within intermediate values (0.086–0.146), while lower values were observed between RCJER, RCRET, TXL and NAC (0.07–0.118).

### 2.4. Ancestral Composition

The dataset used to evaluate the ancestral composition of RC included 10,612 common SNPs and 345 individuals representing Criollo, Iberian taurine, commercial taurine, African taurine, and Indicine breeds (Table S1).

**Figure 3:**
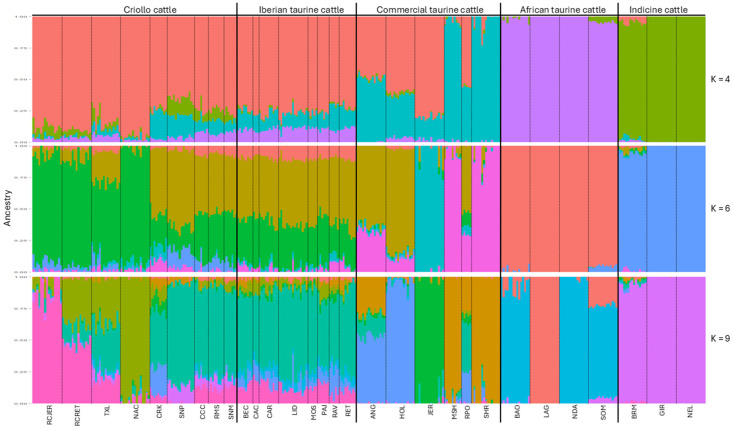
Admixture analysis of Rarámuri Criollo cattle from the Jornada Experimental Range and other Criollo populations. Results for ancestral contributions K = 4, 6, and 9 are presented. RCJER: Rarámuri Criollo cattle from Jornada Experimental Range, RCRET: Rarámuri Criollo cattle from Rancho Experimental Teseachi, TXL: Texas Longhorn, NAC: North American Corriente, CRK: Florida Cracker, SNP: Senepol, CCC: Costeño con Cuernos, RMS: Romosinuano, SNM: San Martinero, BEC: Berrenda en Colorado, CAC: Cachena, CAR: Cardena Andaluza, LID: Lidia, MOS: Mostrenca, PAJ: Pajuna, RAV: Asturiana de los Valles, RET: Retinta, ANG: Angus, HOL: Holstein, JER: Jersey, MSH: Milking Shorthorn, RPO: Red Poll, SHR: Beef Shorthorn, BAO: Baoule, LAG: Lagune, NDA: N’Dama, SOM: Somba, BRM: Brahman, GIR: Gir, NEL: Nelore.

The ancestral contribution to the RCJER herd analyzed for representative K values are presented in Figure 3. The lowest cross-validation error occurred at K = 9 within a range of 1 to 10 (Figure S7). At K = 4, the RCJER herd exhibited a predominant ancestral contribution from Iberian breeds, with minor influences from commercial, Indicine and African cattle. When the number of ancestral populations was increased to K = 6, the influence of Iberian breed decreased, particularly in RCJER, RCRET, and NAC. Additionally, the RC populations and NAC separated from the genetically closest group, TXL. At K = 9, the RCJER and RCRET populations are clearly distinguished from the other Criollo cattle populations, including the TXL and NAC.

A more detailed description of the ancestral contribution to the RCJER herd for K values ranging from 2 to 10 is shown in Figure S8. Briefly, from K = 2 to K = 5, the RCJER and other Criollo cattle populations had strong influence from Iberian cattle with minor contributions from other genetic groups. At higher levels of ancestral populations (K = 6 to K = 10), RCJER maintained a distinct genetic profile, with slight variations in ancestry and limited evidence of introgression from other breeds.

### 2.5. Selection Signature Analysis

To minimize false-positive signals caused by high relatedness, a stringent kinship threshold of 0.177 was applied. Sixty-one RCJER individuals were excluded due to high relatedness, resulting in a final dataset of 90 animals for detecting selection signatures. A total of 53,750 SNPs were used for Tajima’s D and integrated haplotype score (iHS) analyses, while 56,709 SNPs were used for ROH analysis. SNPs identified by at least two methods were considered under positive selection.

The Tajima’s D method identified 327 SNPs across 19 chromosomes as putative candidates for positive selection (Figure S9a; Table S2). The ROH analysis revealed 631 SNPs spanning 16 chromosomes as indicators of potential regions under selection (Figure S9b; Table S2). Lastly, the iHS method detected 326 SNPs across 28 chromosomes showing evidence of positive selection (Figure S9c; Table S2).

In total, 42 SNPs spanning seven chromosomes (1, 2, 6, 7, 13, 18, and 22) were identified by at least two of the three methods used (Table S3).

### 2.6. Identification of Candidate Genes

Candidate regions were defined as genomic intervals spanning ±250 kb flanking each candidate SNP. Gene annotation identified 89 candidate genes associated with a wide range of traits, including milk yield and composition, growth, meat and carcass, reproduction, metabolic homeostasis, health, and coat color (Table 2, Table S3).

**Figure 4:**
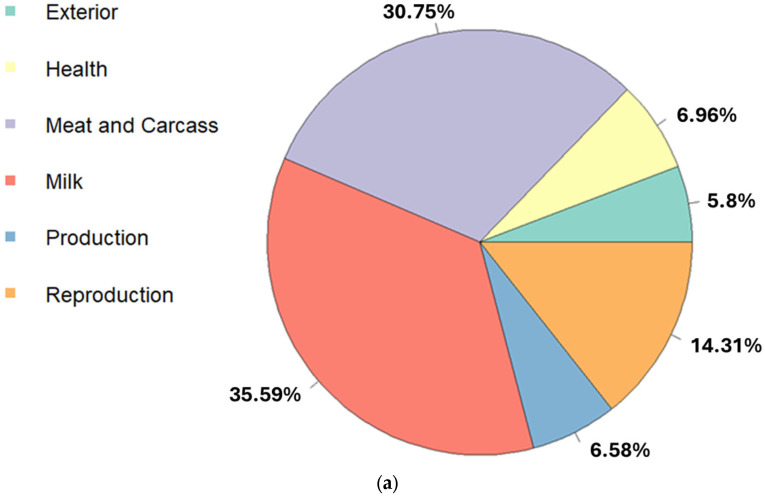

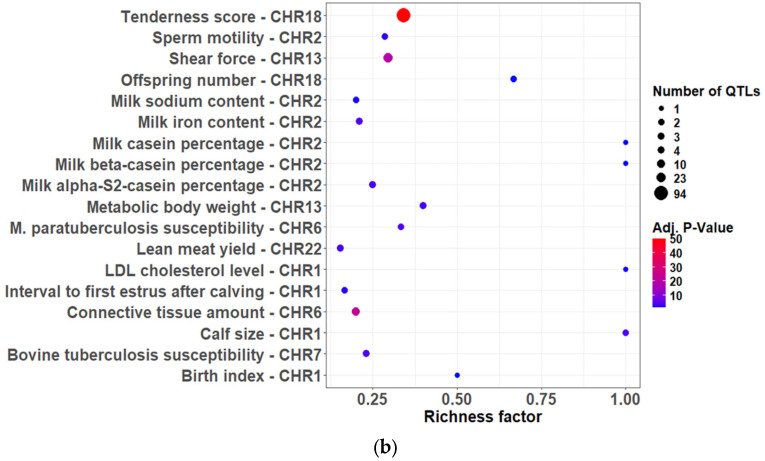
Quantitative trait loci (QTL) annotated in the candidate regions of Rarámuri Criollo cattle from the Jornada Experimental Range. (**a**) The pie chart illustrates the proportion of QTL types. (**b**) The bubble plot displays the 18 enriched QTLs annotated. The color gradient denotes the significance of adjusted *p*-values; the red color indicates more significant enrichment. The area of the circles is proportional to the number of QTLs. The x-axis shows the enrichment factor, calculated as the ratio of the number of QTLs annotated in the candidate regions to the number of each QTL in the reference database.

A total of 517 quantitative trait loci (QTL) were annotated within the candidate regions. The two most frequent QTL types were associated with ‘milk’ and ‘meat and carcass’, while ‘reproduction’, ‘production’, ‘exterior (morphology)’ and ‘health’ were less frequently identified (Figure 4a; Table S4).

A detailed analysis of each QTL type (Figure S10) revealed that the most common traits associated with ‘milk’ were iron content and alpha casein content. For ‘meat and carcass’, the most frequently annotated traits were tenderness score and shear force. Regarding ‘reproduction’ and ‘production’ types, sperm motility and conception rate were the most common in the former, while body weight and body weight gain were more frequently annotated in the latter. For ‘health’, the most frequent traits were bovine tuberculosis susceptibility and tick resistance, whereas white spotting and feet and leg conformation were the most common traits associated with the ‘exterior’ QTL type.

The enrichment analysis identified 18 significant traits associated with the six QTL types mentioned above (Figure 4b; Table S5). Among these, tenderness score, shear force, and connective tissue amount were the most enriched traits.

## 3. Discussion

Rarámuri Criollo cattle are considered resilient and highly adaptable to dry and hot environments due to their historical exposure and adaptation to the harsh conditions of the low- and high-Sierra Tarahumara region (Copper Canyon) in southwestern Chihuahua, Mexico. These cattle have developed primarily under natural selection, enabling them to thrive in harsh environments where other breeds may not [11,12,14]. Since its introduction to the JER, the RC herd has been managed to preserve and evaluate their hardiness in the desert conditions of the southwestern USA [8,15]. The present study characterizes the genetic diversity, ancestral composition, and selection signatures of the RCJER cattle of the USDA-ARS, originating from the isolated herds in the canyons and valleys of the low Sierra Tarahumara.

Heterozygosity values of the RCJER herd were within the range reported for several Criollo cattle populations in South America [29,30,31] and North America [5,32]. Consistent with these heterozygosity values, the RCJER exhibited a moderate F_ROH_ inbreeding of 11.5%. This level was higher than those reported for Criollo cattle populations in Argentina, Bolivia, Peru (3–7.6%) [31] and Colombia (1.1–1.5%) [30], but lower than the inbreeding level of 14% observed for Uruguayan Criollo cattle [31]. Similar to the RCJER herd, the Uruguayan Criollo cattle population was founded with 35 animals and has remained isolated for over 80 years [33], which may explain the higher F_ROH_ in both populations. Additionally, the RCJER population size is smaller than most of the other Criollo cattle populations studied.

The length of ROH segments and the F_ROH_ within these segments were also calculated. The length of a ROH segment serves as an indicator of the timing of the inbreeding events [34]. Short segments (≤4 Mb) accounted for approximately 84% of all identified ROH in RCJER cattle, suggesting this population has predominantly experienced ancient inbreeding dating back 20–25 generations [34].

The historical Ne estimated by the SNeP and GONE programs showed a declining trend over generations, with a value of 33 in the most recent generation. This value is below the recommended level between 50 [35] and 100 [36,37] for maintaining genetic diversity and the overall health of animal populations. Similar to the RCJER herd, Criollo cattle populations from South and North America have exhibited a consistent reduction in Ne [9,31,38,39], with values ranging from 4.8 to 39.8 for the most recent generation of Criollo cattle from Argentina, Bolivia, Peru, and Uruguay [31]. In contrast, other studies have reported higher Ne in Criollo cattle from Colombia (Ne = 123) [38] and Mexico (Ne = 72) [39].

The low decline in genetic diversity observed in the RCJER herd, as evidenced by the heterozygosity and inbreeding levels, may be attributed to the relatively low selection pressure these animals have experienced under the JER conservation program, which included additions over recent years of a few new bulls imported from the Sierra Tarahumara region. However, the low estimated Ne highlights the need to implement measures to increase the genetic diversity of the RCJER population. In the coming years the program is expected to import bulls from the Sierra Tarahumara region.

To fully understand the genetic structure of the RCJER herd, no individuals were excluded based on high relatedness. The original introduction of animals to the JER involved hand-selected individuals purchased from families with small herds located within a 10 km radius [8]. As expected, the PCA and DAPC indicated low population stratification, with no obvious grouping and overlapping of the clusters identified by DAPC. This pattern suggests a shared genetic background among different subfamilies, likely explained by the original selection of animals from different small herds owned by members of the Tarahumara community in 2005. The mean kinship coefficient near zero in the RCJER indicates that most of the individuals are unrelated [28]. Furthermore, this value is below the threshold of 0.1, which is recommended to avoid genetic inbreeding depression in the next generation [28,40]. This information provides a valuable theoretical basis for conserving the RCJER population, for example, by designing breeding plans or selecting specific donor individuals for establishing frozen semen banks or embryo transfer plans to preserve different sublineages or subfamilies.

The genetic relationship between the RCJER herd and other Criollo cattle populations was evaluated using PCA, DAPC, and F_ST_ distance. All three methods, also supported by the admixture results, clearly distinguished the RCJER herd from the other Criollo cattle populations.

As expected, both RC populations, originating from JER and RET, were closely related. Although both herds share the same origin, they developed from different ecological and geographic regions. The RCJER cattle originated from cattle raised in the deep canyons and valleys of the Copper Canyon region, whereas the RCRET cattle originated from herds raised at higher altitudes of the Copper Canyon region [41]. Additionally, both populations have been managed as closed herds and exposed to different environmental conditions since their establishment approximately 20 years ago [20]. The RCJER herd developed in the hot, arid, and relatively flat Jornada del Muerto basin in southwestern New Mexico, USA, whereas the RCRET herd was developed in the semiarid and rugged juniper woodlands in the eastern Sierra Madre foothills, Mexico [20]. Different founder animals, varying natural and management selection, and geographic isolation likely contributed to this genetic differentiation [42]. Furthermore, genetic differentiation is possible between the RCJER and RCRET and the original populations that remain isolated in the Copper Canyon region.

The RCJER population clustered with the RCRET, NAC, and TXL populations, which is consistent with previous studies reporting a close relationship between Mexican Criollo cattle and TXL [3,4,6], as well as between NAC and TXL [43]. Criollo Cattle were introduced to North America through the Mexican port of Veracruz, expanding across Mexico and reaching the region that is known today as New Mexico and Texas, USA [12]. Consequently, TXL, Corriente, and Mexican RC cattle may represent a historical pathway for cattle dispersal and development in North America [4].

The admixture analysis revealed, as expected, a strong Iberian influence in the RCJER population, along with minor contributions from commercial, African, and Indicine breeds. This analysis suggests that the RCJER population has not experienced significant introgression from genetic groups other than Iberian breeds, which is consistent with findings reported for other Criollo cattle groups [4,5,6,44]. As the K values increased, the RCJER population maintained a distinctive genetic profile, showing similarity to the RCRET population up to K = 6, which indicates a shared ancestry. At K = 9, the admixture results aligned with the PCA and DAPC plots, revealing a differentiation between the two RC populations. These findings support the previous discussion based on the results of PCA, DAPC, and F_ST_ genetic distance.

Three statistical methods (Tajima’s D, ROH islands, and iHS) were applied to identify genomic regions potentially under selection in the RCJER herd. The annotated genes are associated with traits such as milk yield and composition, reproduction, growth, meat and carcass, body conformation, color, health and metabolic homeostasis. Consistent with these findings, most QTL annotations corresponded to the aforementioned traits, including ‘milk’, ‘meat and carcass’, and ‘reproduction’, among others. Additionally, the QTL enrichment analysis was conducted to obtain unbiased identification of significant QTLs.

Numerous candidate genes associated with milk composition in livestock were under positive selection in the RCJER herd. Most of the genes are linked to fat, protein, and mineral composition, including *TRNAC-GCA*, *TRNAW-CCA* [45,46], *MRPL3*, *NUDT16*, *NEK11* [47,48], *ALPL* [49,50], *CLINT1* [51,52], *ACSS1* [53,54], *TRNAC-ACA* [46,55], *SMOX* [56], and *ADRAD1D* [57]. Consistent with the gene annotation, most of the annotated QTLs were associated with milk composition, while the enriched QTLs were related to casein and mineral content. These findings suggest that RCJER cows may have adapted to produce milk with high components in nutrient-limited environments, although the milk yield and composition of RC have not yet been characterized.

Several candidate genes under selection in RCJER are linked to body conformation and growth. These include *CPNE4* [58,59], *LEKR1* [60], *HSPG2* [61,62], *TRNAC-GCA* [63], *SYNDIG1* [64], *TRNAS-GGA* [65], and *TRNAW-CCA* [66] which have been associated with body weight and growth. Interestingly, a group of identified candidate genes, including *BMP2* [67], *ALPL* [68,69], *EVC*, and *EVC2* [70,71] play important roles in bone morphogenesis and skeletal development. Particularly, the *ALPL* gene is associated with early skeletal maturity in heifers [68,69], suggesting rapid development and maturity in RC cattle.

In terms of meat and carcass quality, several genes are associated with fat deposition and marbling, including *HSPG2* [72,73], *STK32B* [64,74], *SYNDIG1* [64], *ACSS1* [75], *PYGB* [76], *ABHD12* [77], *TRNAS-GGA* [78], *ATRN* [79,80], *ANKRD16* [81], *BMP2* [82,83], and *RAP1GAP* [84]. Consistent with these findings, QTLs related to tenderness score and shear force were enriched. Additionally, genes associated with muscle growth, such as SMOX [85], *BMP2* [86], *EIF4G3* [87], as well as QTL for lean meat yield, were identified. Limited studies have shown that rangeland-raised RC steers can reach marketable weights by 30 months of age, while producing a carcass that is typical of that for grass-finished beef [8,88,89]. These findings suggest that meat from RC cattle raised and finished on rangeland may represent a local alternative to conventional beef supply chains [88].

Candidate genes related to reproduction traits were also identified. For female reproduction, the genes *CLINT1*, *LSM11*, *THG1L*, and *TRNAW-CCA* [90,91] are linked to age at first calving and first calving interval, both of which are key indicators of puberty and reproductive efficiency in livestock herds. In relation to these traits, two QTLs associated with the interval to first estrus after calving were enriched. Additionally, the genes *SPG2* [92] and *CDC25B* [93] play important roles in oocyte meiosis and follicular growth and differentiation, respectively. Finally, the *TRNAC-GCA* gene has been associated with high fecundity in sheep [94]. Mexican Criollo cattle have been relegated to harsh environments and low-input production systems, which may have contributed to adaptive mechanisms enabling them to reproduce under limiting environmental conditions [10,12,42].

In terms of male reproduction, the *TRNAS-GGA* gene is associated with sperm viability in cattle [95,96,97], while *TRNAC-GCA* has been shown to enhance sperm production and quality in buffalo, dairy and beef cattle [95], as well as sperm quality in goats [98]. However, the *RF00026* gene [99] and two sperm motility QTLs [100] have been linked to testicular hypoplasia and poor sperm motility, respectively. Therefore, close and frequent monitoring to characterize these traits in RC herds is recommended.

Rarámuri Criollo cattle are characterized by low birth weight [11]. Records from the JER indicated a mean birth weight of 21 kg, while the RET at the University of Chihuahua reported birth weights ranging from 24 to 27 kg [101]. An early study reported a birth weight of 17.5 kg in small herds raised in the low Sierra Tarahumara [13]. Due to this low birth weight, de Alba Martinez [11] suggested that dystocia is likely nonexistent in RC. Consistent with these findings, genes associated with reduced birth weight, *TRNAS-GGA* [65,66], *CCNL1*, and *LEKR1* [102,103] were identified as putative genes under selection in the RCJER herd. Furthermore, QTLs related to calf size and offspring number (twinning rate) were enriched, aligning with the gene annotation.

This study also identified genes and QTLs associated with metabolic homeostasis and health. The *ECE-1* gene plays an essential role in maintaining oxygen homeostasis [104,105] and has been linked to adaptation to high altitudes in cattle [106]. Additionally, *ECE-1* has been associated with oxidative stress responses [107]. Regarding health, the *MAVS* gene encodes a protein that enhances antiviral immunity through various mechanisms [108,109,110]. *C13H20orf194* [111] and *HSPG2* [112] are potentially associated with hoof health status, with *HSPG2* specifically linked to the healing process in footrot in sheep [112]. Furthermore, several QTLs related to tuberculosis and paratuberculosis were enriched in the RCJER population. Given that RC cattle have been historically relegated to impoverished conditions with limited animal nutrition and health management [10,12,42], the development of survival mechanisms and disease resistance is expected in the RCJER.

Criollo cattle breeds, including RCJER, exhibit a wide variety of coat colors and patterns [10,16]. Several candidate genes associated with coat color were identified in the RCJER herd. For example, *CYFIP2* influences coat color variation (brown, white and black) in Sumatran native cattle [113]. This study also identified *ATRN* as a candidate gene, which is known for its role in normal pigment production [114] and is part of a group of genes related to changes in coat color pattern as cattle age from calves to adults, as well as in response to environmental factors [115]. Additionally, *ATRN* contributes to eye area pigmentation in cattle [116], which may reduce susceptibility to bovine ocular carcinoma in regions like the Chihuahuan Desert, where cattle are exposed to high levels of solar radiation.

Since the sampled animals were from a single herd, caution should be exercised when interpreting this data. Additionally, the RCJER herd has been managed in isolation for nearly 20 years; as a result, it is possible that due to local selection, genetic drift, or climatic conditions, RCJER has diverged genetically from the RC populations remaining in the Tarahumara region. Therefore, the findings of this study should not be interpreted as a comprehensive genetic characterization of the RC cattle as a whole.

In conclusion, RCJER may represent a unique genetic resource, as previously suggested by Anderson et al. [8] and de Alba Martinez [11]. The detection of selection signatures associated with valuable adaptation and performance traits indicates that RCJER cattle may be highly resilient and adaptable to harsh environmental conditions while retaining positive productive and reproductive attributes. However, these findings need to be supported by additional genome-wide association studies, transcriptome profiling, fine mapping, among other studies. Further efforts should be made to enhance the genetic diversity of the current population and ensure the long-term conservation and expansion of this distinctive Criollo cattle.

## 4. Materials and Methods

### 4.1. Sampling, Genotyping and Quality Control

The RC population evaluated in this study belongs to the nucleus maintained at the USDA-ARS JER (https://jornada.nmsu.edu, accessed on 9 May 2025) located in southern New Mexico, USA, within the northern Chihuahuan Desert ecoregion. The breeding program has operated as a closed genetic herd with random mating since its establishment in 2005 [8]. As part of the ongoing efforts to conserve and expand the RC, a group of JER cattle were acquired in 2019 and 2022 by The Nature Conservancy’s Dugout Ranch (DR) located in the Colorado Plateau in southeastern Utah, USA (https://www.nature.org/en-us/get-involved/how-to-help/places-we-protect/dugout-ranch/, accessed on 29 October 2024).

A total of 152 ear tissue punches (Tissue Sampling Unit, Allflex, NJ, USA) were collected from JER (*n* = 118) and DR (*n* = 34). The entire existing population was sampled in both herds. The samples from DR consisted of ear tissues from mature cows recently introduced from JER; therefore, both herds were analyzed as a single RC population. The resulting dataset is referred to as RCJER (Rarárumi Criollo from the Jornada Experimental Range) throughout the article.

Genotyping was performed at the Genetic Visions-ST^TM^ laboratory (Middleton, WI, USA) using the VM2 SNP array, which contains 63,683 SNPs mapped to the bovine genome assembly ARS-UCD1.2/bosTau9. SNPs located on sex chromosomes and those with unknown coordinates were excluded. Quality control performed with PLINK v.1.9 [117] is detailed in each section below.

### 4.2. Genetic Diversity

Quality control was conducted following the FAO’s recommendations for genomic characterization of animal genetic resources [118]. The following parameters were applied: missing call rate per SNP (--geno < 0.05), minor allele frequency (--maf > 0.01), individual missingness (--mind < 0.05), deviation from Hardy–Weinberg equilibrium (--hwe 0.000001), and linkage disequilibrium (LD) pruning (--indep-pairwise 50 5 0.2). Although it is advisable to remove related animals, in this study, animals with high relatedness (i.e., parents–offsprings or full siblings) were retained to enable a comprehensive analysis of the population dynamics within the RCJER herd [117].

The genetic diversity of the RCJER population was evaluated using H_O_, H_E_, Ne, and F_ROH_. H_O_ and H_E_ were calculated using PLINK v1.9 software. For ROH, the same quality control criteria as in previous sections were applied, except that no MAF or LD pruning was performed, following the guidelines of Meyermans et al. [27]. Pruning for low MAF can ignore large homozygous genomic regions, while LD pruning leads to a reduction in SNP density in homozygous regions [27]. ROH were identified using the consecutive runs method [119] implemented in the R package detectRUNS v0.9.6 [120]. The following parameters were used: (i) maximum gap between consecutive homozygous SNPs: 1000 kb; (ii) minimum ROH length: 250 kb; (iii) maximum number of opposite genotypes: 1; (iv) maximum number of missing genotypes: 1; and (v) minimum number of SNPs in an ROH: 22, calculated using the formula proposed by Lencz et al. [121] and modified by Purfield et al. [32]. The number of ROH per individual was calculated and classified into five length categories: >1–2 Mb, >2–4 Mb, >4–8 Mb, >8–16 Mb and >16 Mb.

The genomic inbreeding coefficient F_ROH_ was calculated as the ratio of the total length of all ROHs for each individual to the total autosomal SNP coverage, which corresponds to 2.51 Gb. F_ROH_ was also computed for different length categories (>1–2 Mb, >2–4 Mb, >4–8 Mb, >8–16 Mb and >16 Mb).

The Ne was calculated using two different software programs, SNeP v1.1 [122] and GONE [123]. Both programs are LD-based methods but SNeP assumes a linear relationship between Ne and the number of generations, providing better estimations for past generations. GONE provides more complex demographic histories and is particularly accurate for recent generations [123]. SNeP was run with default settings, except for the sample size correction and Sved and Feldman’s mutation rate modifier [124], as described in Pitt et al. [9]. GONE was used with the default settings. Results from both analyses were plotted using the R package ggplot2 v3.5.0 [125].

### 4.3. Population Structure and Genetic Relationships

By integrating population structure and genetic relationship analyses, it is possible to examine genetic or family substructures, inferring which individuals are more closely related and likely to descend from a same family lineage [126,127].

Quality control was conducted following the FAO’s recommendations, as described in Section 4.2. PCA was performed using PLINK v1.9 and visualized with the R package ggplot2 v3.5.0. DAPC analysis was performed using the R package adegenet v2.1.7 [128]. The most likely number of K (ranging from 1 to 10) was selected using the K-means method. The optimal number of principal components was determined through cross-validation method. To assess the degree of relatedness, the KING-robust method [38] was implemented in PLINK v2.0 [129].

### 4.4. Relationship with Other Criollo Cattle Populations

The relationship between RCJER and other Criollo cattle populations was evaluated using PCA, DAPC, and pairwise F_ST_ genetic distance.

The RCJER dataset was combined with six Criollo cattle populations described by Pitt et al. [9]: CCC, RMS, SNM, CRK, Senepol, and TXL. Additionally, animals from the RCRET population in Chihuahua, Mexico, and from an NAC population in New Mexico, USA, were included. Samples were generously provided by the Universidad Autónoma de Chihuahua and New Mexico State University, respectively. Genotyping of RCRET and NAC animals was conducted at Genetic Visions-ST^TM^ (Middleton, WI, USA) using the VM2 SNP array (63,683 SNPs).

To address the imbalance in sample sizes between populations, the random sampling procedure implemented in the R package BITE v1.2 [130] was applied to select a maximum of 15 representative individuals per Criollo cattle population (Table S1). After quality control, a total of 10,612 SNPs common to all datasets were retained.

The PCA was performed using PLINK v1.9 and visualized with the R package ggplot2 v3.5.0. DAPC analysis was conducted using the R package adegenet v2.1.7. The most likely number of K, ranging from 1 to 15, was selected using the K-means method. The optimal number of principal components was determined through cross-validation method.

The pairwise F_ST_ genetic distances between populations were calculated using the R package StAMPP [131] with 1000 bootstraps and visualized in a heatmap built with the R package ggplot2 v3.5.0.

### 4.5. Ancestral Composition

The contribution of taurine and Indicine ancestry to the genomic composition of the RCJER population was evaluated using a maximum likelihood model implemented in the ADMIXTURE v1.3 software [132]. The Criollo cattle dataset was merged with the dataset described by Pitt et al. [9], which includes (i) Iberian taurine, (ii) commercial taurine, (iii) African taurine, and (iv) Indicine breeds. A maximum of 15 representative individuals per population were selected using the R package BITE v1.2 (Table S1). Ancestry was tested for a range of population K from 1 to 10. The best-fitting K value was estimated using the fivefold cross-validation procedure implemented in ADMIXTURE v1.3. Furthermore, a range of values were explored as suggested [133]. The graphical representation of admixture patterns was generated using the R package ggplot2 v3.5.0.

### 4.6. Selection Signature Analysis

To minimize false-positive signals caused by high relatedness among individuals [134], the KING robust kinship estimation method [28] was applied using the --king-cutoff flag in PLINK v2.0 with a stringent value of 0.177 [129].

The use of multiple analytical approaches has been suggested to increase the reliability of detection selection signature analyses [135]. In the present study, three methods were used: Tajima’s D statistics (based on site frequency spectrum), ROH (based on reduced local variability), and iHS (based on linkage disequilibrium).

Tajima’s D values [136] were calculated using VCFtools v0.1.16 [137] with a window size of 500 kb. The bottom 1% of Tajima’s D values were considered as genomic regions under selection [138].

The ROHs were identified using the consecutive runs method implemented in the R package detectRUNS v0.9.6 as described in Section 4.2. The top 1% of ROH frequencies were considered as regions under positive selection [139].

The iHS values [140] were calculated using the R package rehh v3.2.2 [141]. Genotype phasing was conducted with Beagle v5.4 software using the default settings [142]. The top 1% of iHS values were considered as regions under positive selection [138].

Manhattan plots were created using the R package qqman v0.1.9 [143] and only SNPs simultaneously identified by at least two methods were considered under positive selection.

### 4.7. Identification of Candidate Genes

Candidate regions were defined as those located within a span of ±250 kb from each candidate SNP [144,145]. Genes were annotated using the BovineMine Database v1.6 [146] with coordinates based on the ARS-UCD1.2 genome assembly.

The QTL annotation was performed using the R package GALLO v1.1 [147] with the annotation file derived from the Animal Genome cattle QTL database Release 55 [148]. A chromosome-based QTL enrichment analysis was conducted, and *p*-values were adjusted for multiple testing corrections using the false discovery rate (FDR ≤ 0.05).

Additionally, a comprehensive literature search was conducted to explore the biological functions and phenotypes affected by the annotated genes.

## Figures and Tables

**Table 1:** Distribution of runs of homozygosity (ROH) and inbreeding coefficients based on ROH (F_ROH_) across different length classes in Rarámuri Criollo cattle from the Jornada Experimental Range.

Class (Mb)	ROH		F_ROH_		
	Number	%	Mean	SD	Range
>1–2	7620	63.25	0.106	0.062	0.023–0.402
>2–4	2533	21.03	0.078	0.064	0.004–0.382
>4–8	909	7.55	0.062	0.062	0.002–0.367
>8–16	574	4.76	0.068	0.056	0.003–0.358
>16	411	3.41	0.048	0.041	0.006–0.274

**Table 2:** Genomic regions under selection and associated candidate genes in Rarámuri Criollo cattle from the Jornada Experimental Range.

Chr	SNPs	Position (bp)	Candidate Genes	Trait
1	1	110,375,430–110,875,430	*RF00026*, *CCNL1*, *LOC112447734*, *LEKR1*, *TRNAW-CCA*, *LOC101902535*	reproduction
1	6	137,774,984–138,733,932	*CPNE4*, *MIR2288*, *MRPL3*, *LOC104971058*, *NUDT16*, *NEK11*, *RF00026*	milk, growth
2	8	130,797,318–131,607,803	*LOC101905607*, *LOC515042*, *CELA3B*, *LOC100847958*, *LOC789612*, *HSPG2*, *LDLRAD2*, *USP48*, *RAP1GAP*, *TRNAC-GCA*, *ALPL*, *RF00026*, *LOC101906756*, *ECE1*, *LOC112443420*, *LOC112443419*, *EIF4G3*	milk, growth, meat, reproduction
6	1	103,360,904–103,860,904	*CRMP1*, *EVC*, *EVC2*, *RF00026*, *TRNAG-CCC*, *STK32B*	meat, bone development
7	4	69,034,944–69,695,703	*CYFIP2*, *NIPAL4*, *ADAM19*, *SOX30*, *THG1L*, *LSM11*, *CLINT1*	milk, coat color
13	5	42,300,967–43,138,553	*SYNDIG1*, *TRNAG-CCC*, *LOC112449290*, *CST7*, *LOC107133049*, *APMAP*, *ACSS1*, *LOC112449375*, *VSX1*, *MIR2285df*, *ENTPD6*, *PYGB*, *ABHD12*, *LOC112449292*, *TRNAC-ACA*, *ANKRD16*, *LOC112449291*, *GDI2*, *FAM208B*, *RF00322*, *ASB13*, *LOC104973792*	milk, meat
13	1	49,119,652–49,619,652	*BMP2*, *LOC104973807*	growth, meat
13	9	50,252,256–51,412,240	*HAO1*, *ADRA1D*, *SMOX*, *LOC104973937*, *RNF24*, *PANK2*, *MIR103A2*, *MIR103-2*, *MAVS*, *AP5S1*, *CDC25B*	meat, health
13	1	51,530,574–52,030,574	*GFRA4*, *ATRN*, *C13H20orf194*, *SLC4A11*, *ITPA*, *DDRGK1*, *LZTS3*	meat, health
18	1	24,264,845–24,764,845	*GNAO1*, *LOC112442287*, *CES5A*, *TRNAS-GGA*, *BREH1*	meat
22	5	13,892,598–14,508,727	*ULK4*, *LOC107131659*, *TRAK1*	neurogenesis

## Data Availability

The original data presented in the study are openly available on the Environmental Data Initiative at https://portal.edirepository.org/nis/mapbrowse?packageid=knb-lter-jrn.200036002.2, accessed on 7 April 2025.

## Supplementary Materials

The following supporting information can be downloaded at: https://www.mdpi.com/article/10.3390/ijms26104649/s1.

## Abbreviations

The following abbreviations are used in this manuscript:

RC	Rarámuri Criollo
JER	Jornada Experimental Range
RCJER	Rarámuri Criollo from the Jornada Experimental Range
HO	Observed heterozygosity
HE	Expected heterozygosity
ROH	Runs of homozygosity
FROH	Genomic inbreeding coefficient based on runs of homozygosity
Ne	Effective population size
PCA	Principal component analysis
DAPC	Discriminant Analysis of Principal Components
K	Cluster
PC	Principal components
DA	Discriminant Axis
iHS	Integrated haplotype score
QTL	Quantitative trait loci
NAC	North American Corriente
TXL	Texas Longhorn
RCRET	Rarámuri Criollo from the Rancho Experimental Teseachi
CRK	Florida Cracker
SNP	Senepol
CCC	Costeño con Cuernos
RMS	Romosinuano
SNM	San Martinero
BEC	Berrenda en Colorado
CAC	Cachena
CAR	Cardena Andaluza
LID	Lidia
MOS	Mostrenca
PAJ	Pajuna
RAV	Asturiana de los Valles
RET	Retinta
ANG	Angus
HOL	Holstein
JER	Jersey
MSH	Milking Shorthorn
RPO	Red Poll
SHR	Beef Shorthorn
BAO	Baoule
LAG	Lagune
NDA	N’Dama
SOM	Somba
BRM	Brahman
GIR	Gir
NEL	Nelore
