# Supplementary material for: Genetic Diversity, Admixture, and Selection Signatures in a Rarámuri Criollo Cattle Population Introduced to the Southwestern United States
Source: Int J Mol Sci. 2025 May 13;26(10):4649. doi: 10.3390/ijms26104649 (PMC12112442; doi:10.3390/ijms26104649)
Supplement: Supplementary file 1 [file ijms-26-04649-s001.zip › Figures S1-S10.pdf]

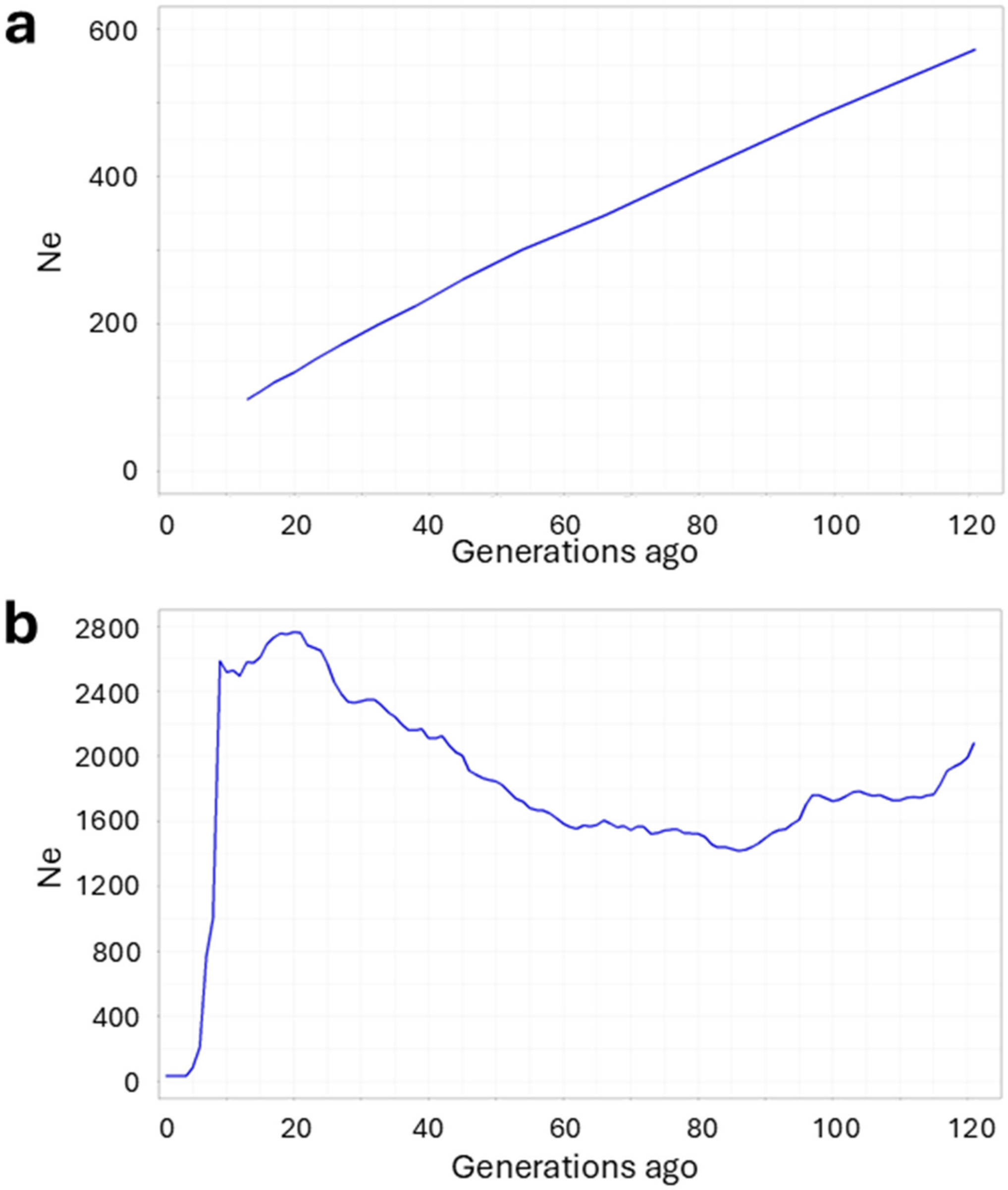

Figure S1. Historical effective population size estimates for Rarámuri Criollo cattle from the Jornada Experimental Range, calculated using the (a) SNeP and (b) GONE software program.

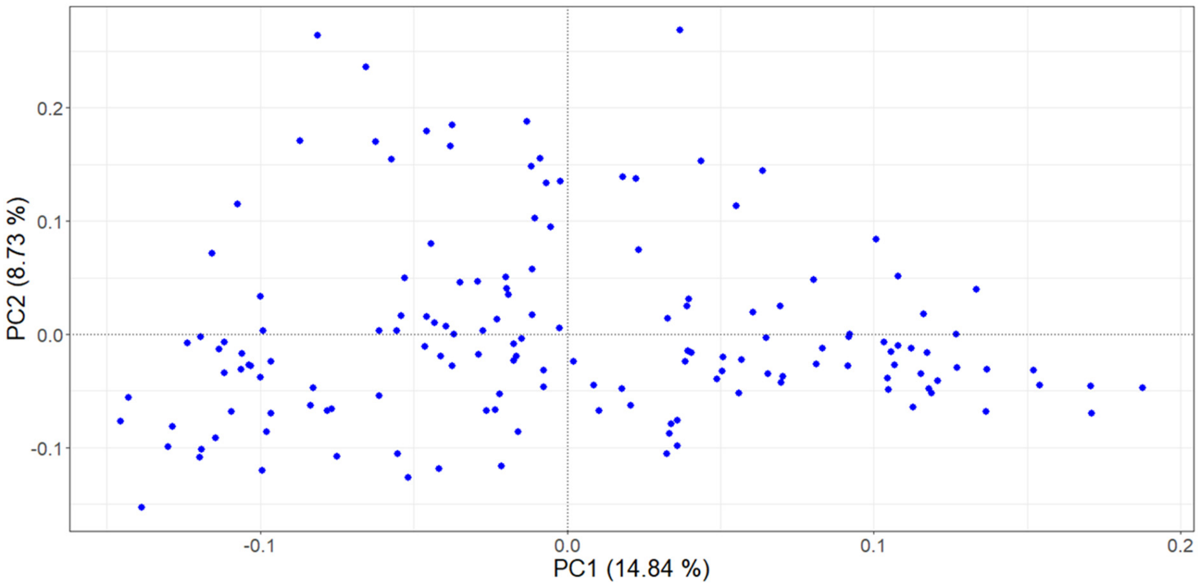

Figure S2. Principal component analysis of Genetic structure of Rarámuri Criollo cattle from the Jornada Experimental Range.

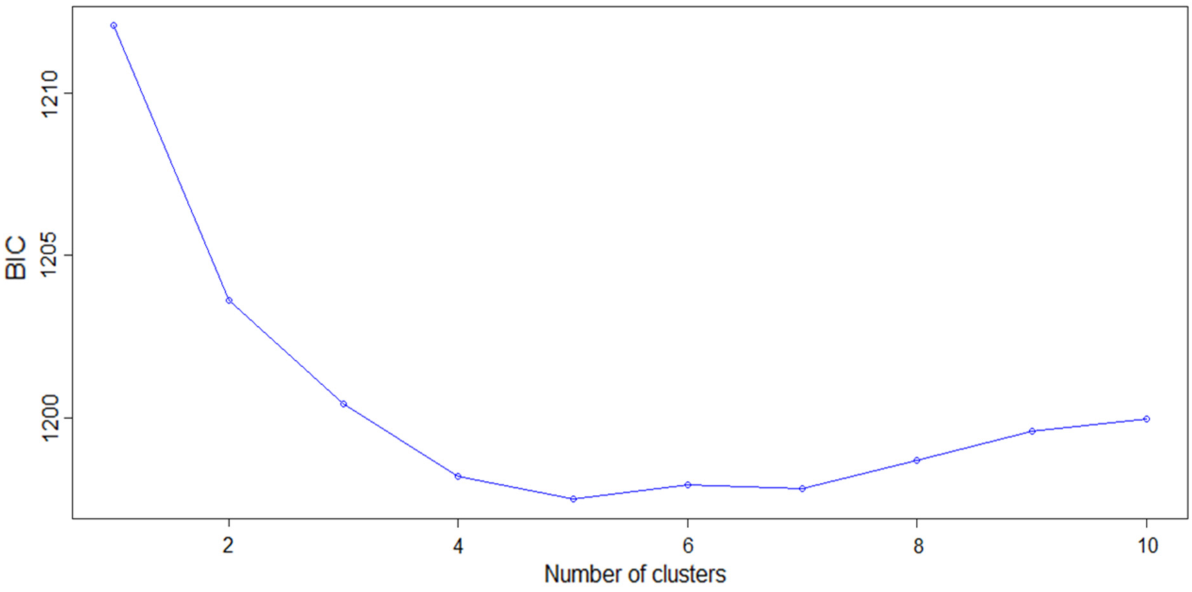

Figure S3. Inference of the number of clusters in Rarámuri Criollo cattle from the Jornada Experimental Range based on K-means algorithm.

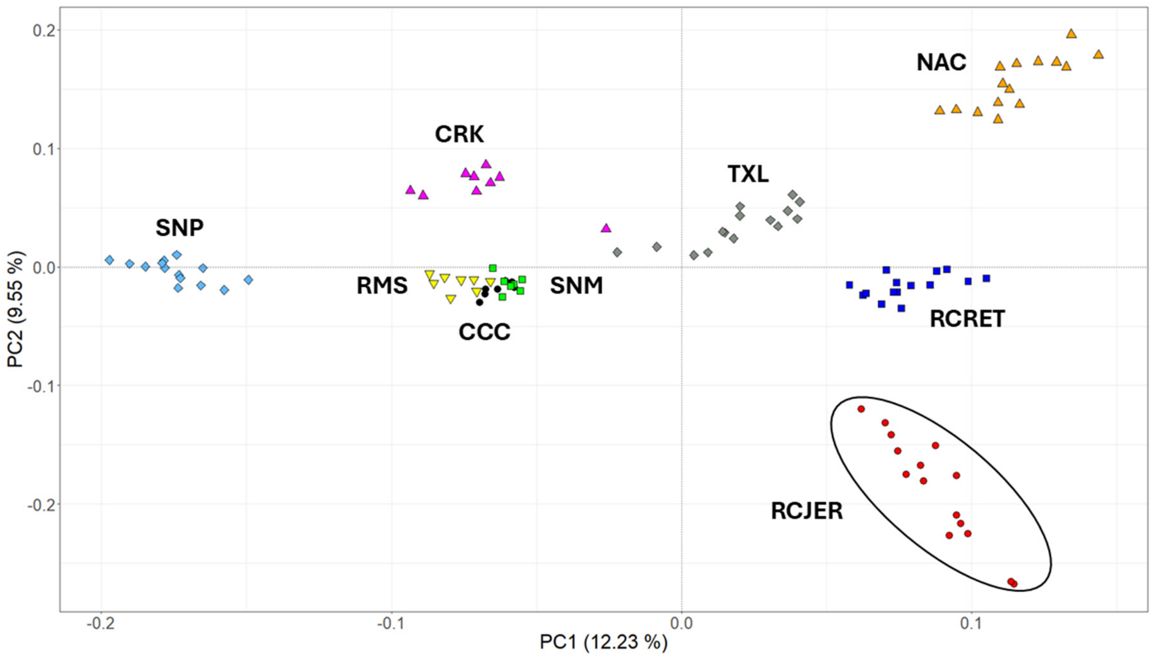

Figure S4. Principal component analysis of nine Criollo cattle populations. Rarámuri Criollo from the Jornada Experimental Range is highlighted with an oval shape. RCJER: Rarámuri Criollo cattle from the Jornada Experimental Range, RCRET: Rarámuri Criollo cattle from the Rancho Experimental Teseachi, TXL: Texas Longhorn, NAC: North American Corriente, CRK: Florida Cracker, SNP: Senepol, CCC: Costeño con Cuernos, RMS: Romosinuano, SNM: San Martinero.

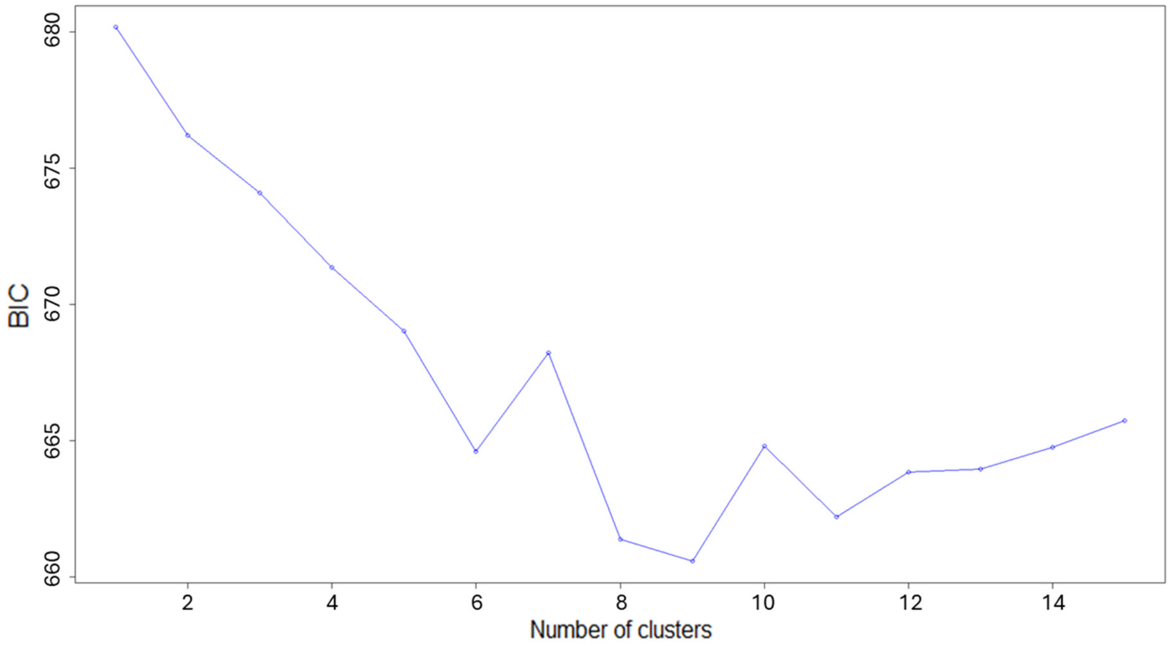

Figure S5. Inference of the number of clusters of nine Criollo cattle populations based on K-means algorithm.

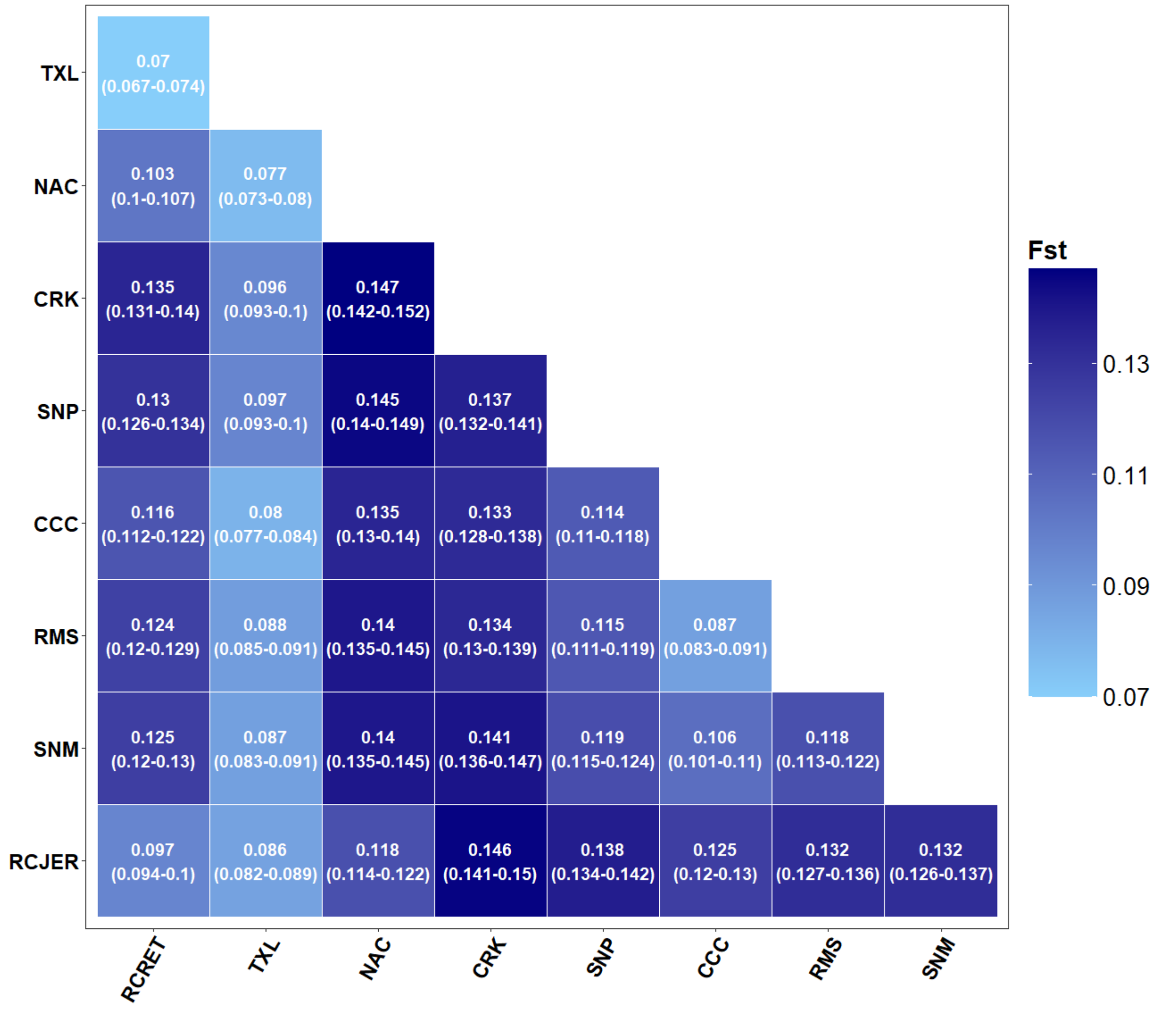

Figure S6. Matrix and heatmap of Fixation Index (F_{ST}) pairwise comparisons among nine Criollo cattle populations. Fixation index followed by a darker blue color indicates greater genetic distance between groups. RCJER: Rarámuri Criollo cattle from the Jornada Experimental Range, RCRET: Rarámuri Criollo cattle from the Rancho Experimental Teseachi, TXL: Texas Longhorn, NAC: North American Corriente, CRK: Florida Cracker, SNP: Senepol, CCC: Costeño con Cuernos, RMS: Romosinuano, SNM: San Martinero.

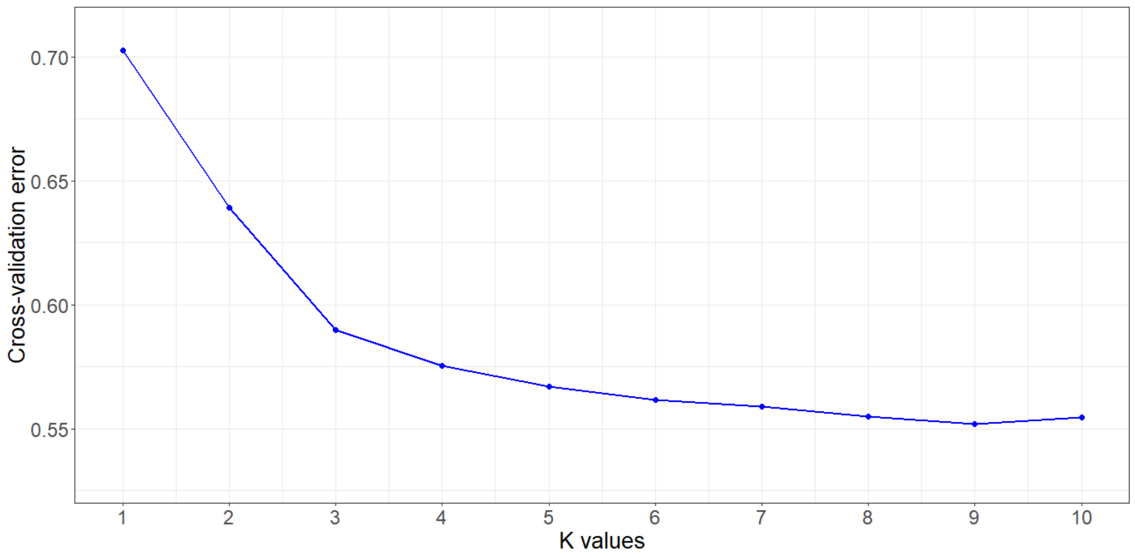

Figure S7. Cross-validation error in the admixture analysis for K varying from 2-10 K = 2 – K = 10.

.

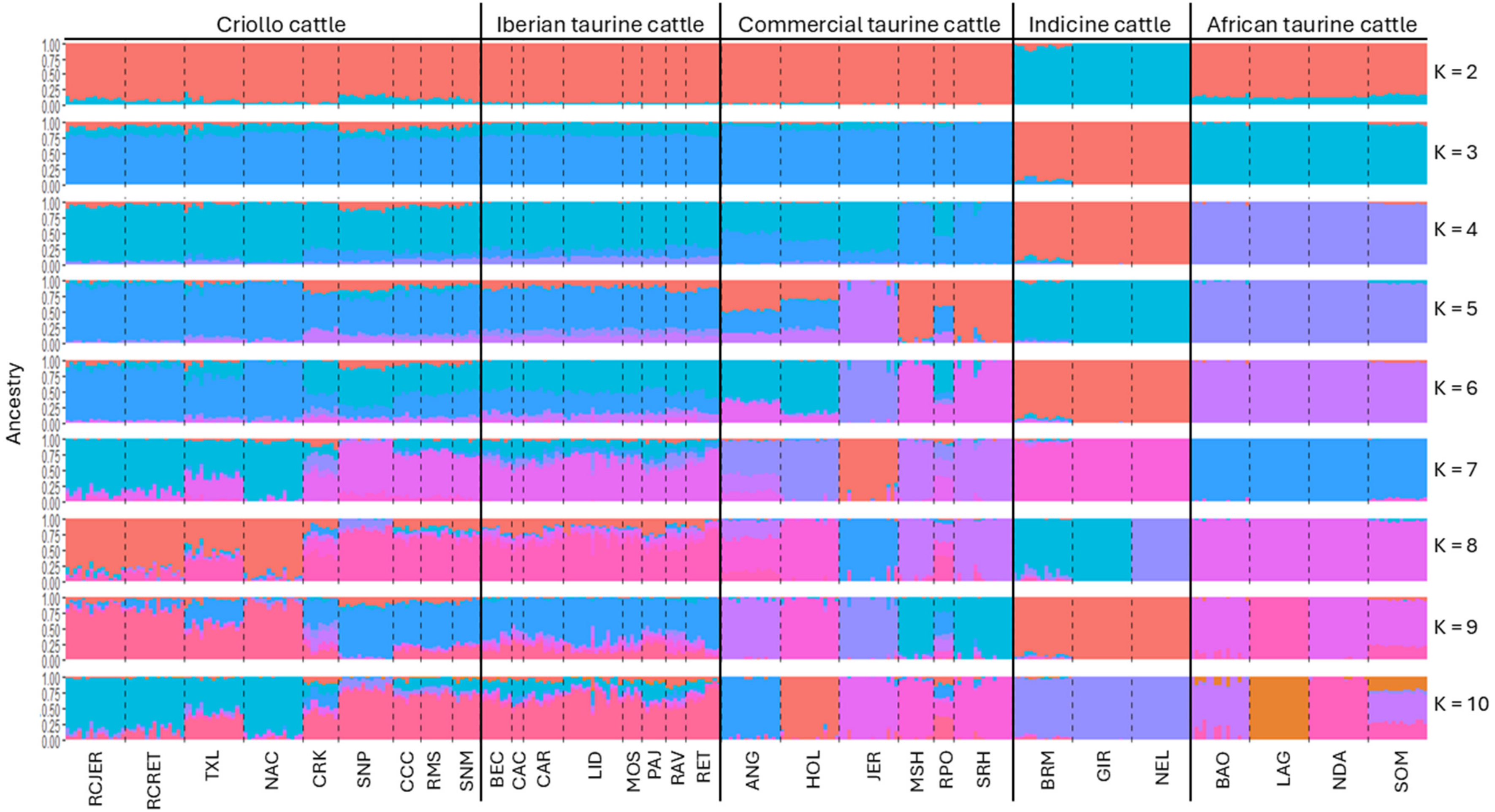

Figure S8. Admixture analysis (K = 2 – K = 10) of nine criollo cattle populations compared to other Iberian, commercial, African and Indicine breeds. RCJER: Rarámuri Criollo cattle from the Jornada Experimental Range, RCRET: Rarámuri Criollo cattle from the Rancho Experimental Teseachi, TXL: Texas Longhorn, NAC: North American Corriente, CRK: Florida Cracker, SNP: Senepol, CCC: Costeño con Cuernos, RMS: Romosinuano, SNM: San Martinero, BEC: Berrenda en Colorado, CAC: Cachena, CAR: Cardena Andaluza, LID: Lidia, MOS: Mostrenca, PAJ: Pajuna, RAV: Asturiana de los Valles, RET: Retinta, ANG: Aberdeen Angus, HOL: Holstein, JER: Jersey, MSH: Milking Shorthorn, RPO: Red Poll, SHR: Beef Shorthorn, BAO: Baoule, LAG: Lagune, NDA: N'Dama, SOM: Somba, BRM: Brahman, GIR: Gir, NEL: Nelore.

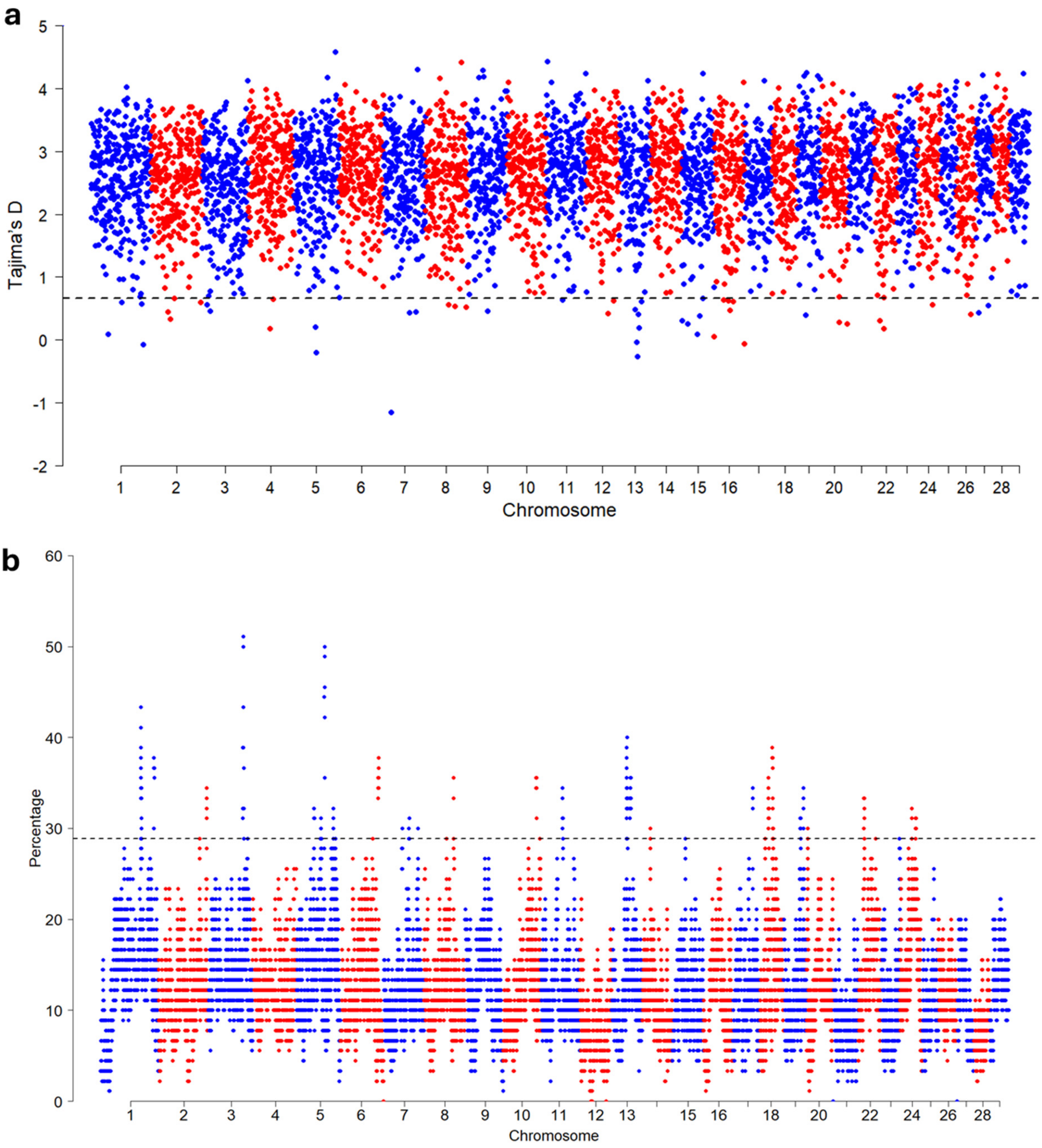

a
Tajima's D
Chromosome
b
Percentage
Chromosome

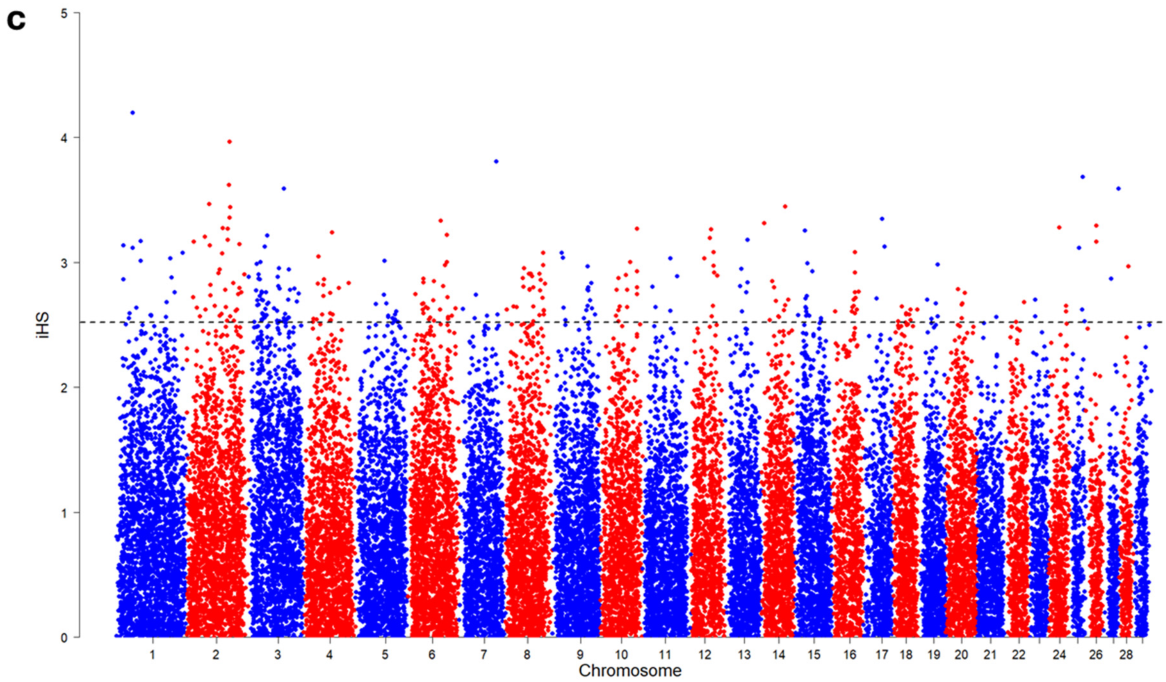

Figure S9. Manhattan plots of genome-wide distribution of selection signatures in Rarámuri Criollo cattle from the Jornada Experimental Range detected using **(a)** Tajima's D statistics, **(b)** runs of homozygosity, and **(c)** integrated Haplotype Scoring. The dotted horizontal line shows the cut-off value to call SNP outliers.

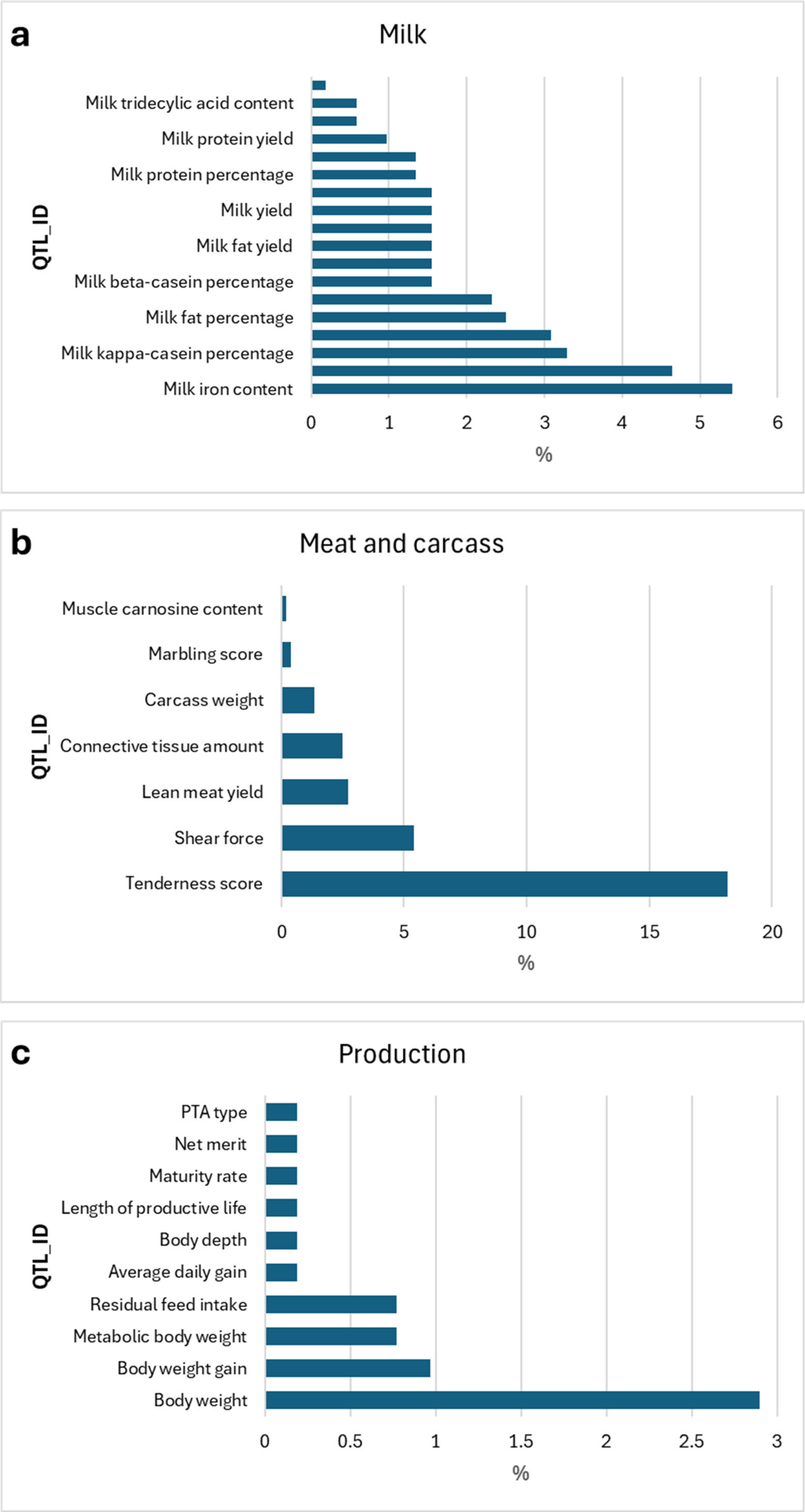
a
Milk
QTL_ID
Milk tridecylic acid content
Milk protein yield
Milk protein percentage
Milk yield
Milk fat yield
Milk beta-casein percentage
Milk fat percentage
Milk kappa-casein percentage
Milk iron content
0 1 2 3 4 5 6
%
b
Meat and carcass
QTL_ID
Muscle carnosine content
Marbling score
Carcass weight
Connective tissue amount
Lean meat yield
Shear force
Tenderness score
0 5 10 15 20
%
c
Production
QTL_ID
PTA type
Net merit
Maturity rate
Length of productive life
Body depth
Average daily gain
Residual feed intake
Metabolic body weight
Body weight gain
Body weight
0 0.5 1 1.5 2 2.5 3
%

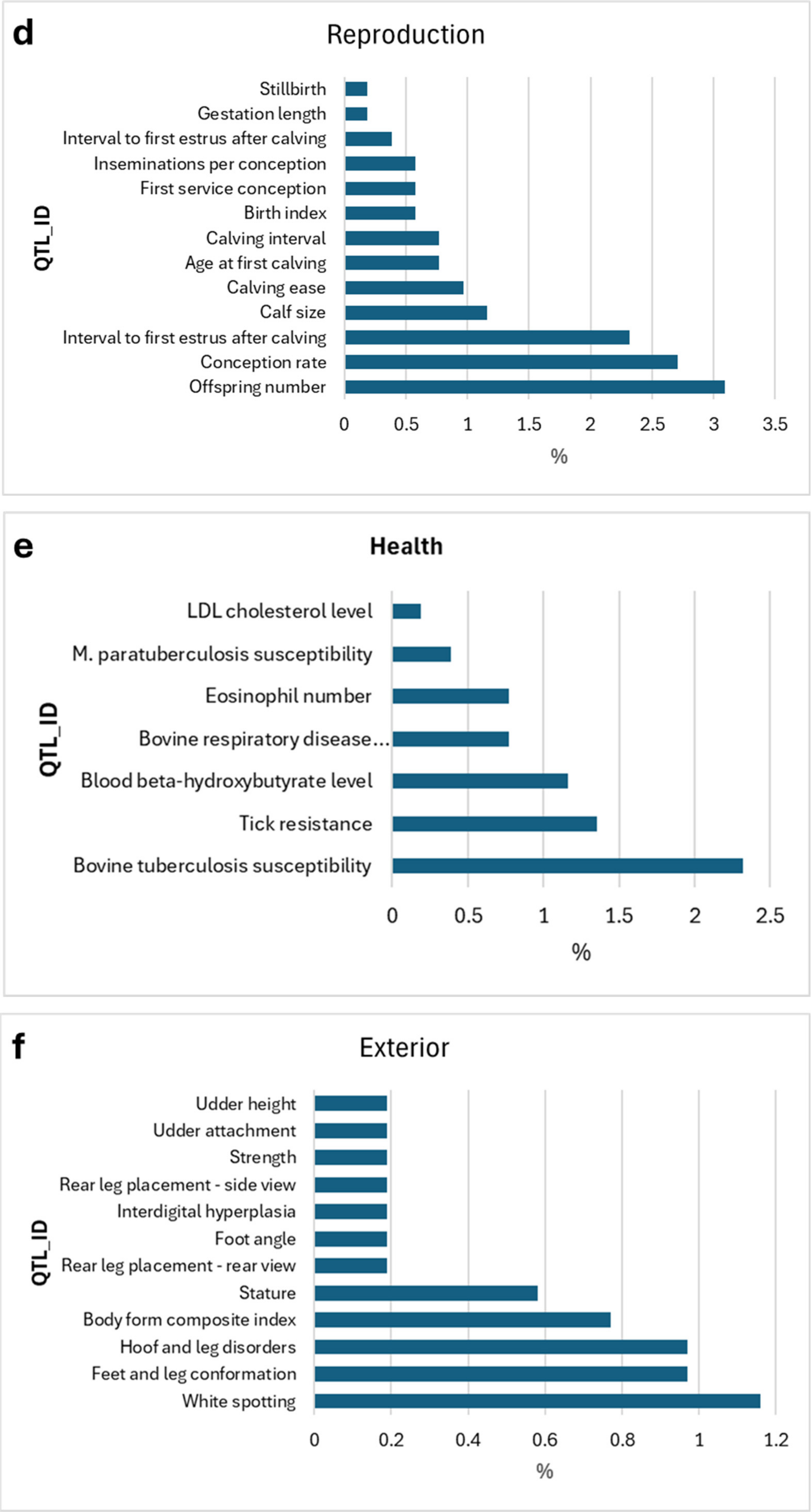
d
Reproduction
QTL_ID
Stillbirth
Gestation length
Interval to first estrus after calving
Inseminations per conception
First service conception
Birth index
Calving interval
Age at first calving
Calving ease
Calf size
Interval to first estrus after calving
Conception rate
Offspring number
0
0.5
1
1.5
2
2.5
3
3.5
%
e
Health
QTL_ID
LDL cholesterol level
M. paratuberculosis susceptibility
Eosinophil number
Bovine respiratory disease...
Blood beta-hydroxybutyrate level
Tick resistance
Bovine tuberculosis susceptibility
0
0.5
1
1.5
2
2.5
%
f
Exterior
QTL_ID
Udder height
Udder attachment
Strength
Rear leg placement - side view
Interdigital hyperplasia
Foot angle
Rear leg placement - rear view
Stature
Body form composite index
Hoof and leg disorders
Feet and leg conformation
White spotting
0
0.2
0.4
0.6
0.8
1
1.2
%

Figure S10. Frequency of QTL presence for (**a**) milk, (**b**) meat and carcass, (**c**) production, (**d**) reproduction, (**e**) health and (**f**) exterior traits in Rarámuri Criollo cattle from the Jornada Experimental Range.
